# Street Dust Heavy Metal Pollution Source Apportionment and Sustainable Management in A Typical City—Shijiazhuang, China

**DOI:** 10.3390/ijerph16142625

**Published:** 2019-07-23

**Authors:** Kui Cai, Chang Li

**Affiliations:** 1 Institute of Geological Survey, Hebei GEO University, Shijiazhuang 050031, China; 2 College of Business Administration, Wonkwang University, 460 Iksandae-ro, Iksan, Jeonbuk 54538, Korea

**Keywords:** heavy metals, source, APCS-MLR, street dust, Shijiazhuang, management

## Abstract

Street dust is repeatedly raised by the wind as a secondary suspension, helping heavy metals therein to enter the human body through the respiratory system, harming human health. A detailed investigation was conducted to determine levels and sources of Cd (cadmium), Cr (chromium), Cu (copper), Pb (lead), Zn (zinc), Ni (nick), and Hg (mercury) contamination in street dust from Shijiazhuang, China. The average concentrations of these metals were: Cd, 1.86 mg·kg^−1^; Cr, 131.7 mg·kg^−1^; Ni, 40.99 mg·kg^−1^; Cu, 91.06 mg·kg^−1^; Pb, 154.78 mg·kg^−1^, Hg, 0.29 mg·kg^−1^; and Zn, 496.17 mg·kg^−1^—all of which were greater than the local soil reference values. The concentrations of the heavy metals were mapped for the three Shijiazhuang ring roads, with the results showing significant differences between each ring. Application of enrichment factors and geoaccumulation indexes showed that there was significant enrichment and accumulation of Cd, Pb, Zn, and Hg. Multivariate statistical analyses showed that Cd, Pb, Zn, and Hg levels were mainly controlled by human activities, while Cr, Ni, and Cu levels were associated with natural sources. Absolute principal component scores with multiple linear regression (APCS-MLR) were applied to facilitate source apportionment. The results showed that the mixed (traffic and industry) group contributed 53.55%, 59.7%, and 62.25% of the Cd, Pb, and Zn concentration, respectively, while the natural sources group contributed 58.01%, 65.09%, and 66.91% of the Cu, Ni, and Cr concentration, respectively. The burning coal group was found to be responsible for 63.38% of the Hg present in the samples. These results provide a useful theoretical basis for Shijiazhuang authorities to address heavy metal pollution management.

## 1. Introduction

Street dust is not only a type of urban pollution in itself, but is also an important medium for the spread of other polluting substances in the urban environment. Due to the influence of industrial production, urban traffic, and industry emission activities, street dust contains many harmful substances, enhancing the risk it represents to human health [1,2,3,4,5]. Generally, soil particles <100 µm in street dust contain more serious environmental hazards and health risks than soil particles >100 µm, and dust or soil particles smaller than 100 µm contain more harmful pollutants and are more likely to stick to human skin [1,6]. Heavy metal pollutants are of most concern, due to their high concentrations in street dust, their capacity for biological enrichment, and their toxic effects on organisms [7,8,9,10]. 

Most of the pollutants carried by street dust have been released recently and locally, and so are a good indication of regional environmental conditions [11,12,13,14]. Street dust is repeatedly raised, settled, and raised again, to a certain height, and residents are inevitably exposed to it directly or indirectly, which allows any heavy metals present easy access to human bodies, through the respiratory system, where they can cause harm [15,16]. In addition, heavy metals are able to degrade, and surface runoff after rainfall washes the street dust, with its entrained load of heavy metals, into the surface water ecosystem where it becomes biologically enriched and can eventually enter the human body through the food chain. This adds another avenue through which the heavy metals in street dust have toxic effects on humans by respiratory and dermal contact after more bioaccumulation [15,16,17].

Research has shown that heavy metals are enriched in the street dust of large and medium-sized cities globally (Table 1), including those in Asia [17,18,19,20,21,22,23,24,25,26], North America [27], Europe [28,29], and Africa [30,31,32]. Shijiazhuang, as a representative city, has numerous traditional Chinese medicine manufacturing factories, chemical raw materials plants, coking factories, textile factories, steel foundries, and leather and fur industries, which together account for 45.1% of its GDP. Heavy industry is intensive and environmental pollution here is more serious than in other parts of the world, particularly air pollution. Shijiazhuang is also prone to sandstorms, which cause secondary dust pollution; the city also has high static wind frequency, poor diffusion ability, and a clear urban heat island effect. These characteristics combine to mean that heavy metal levels in atmospheric particles, urban soil, and street dust are more serious than normal.

In recent years, multivariate statistical techniques—such as the correlation analysis [1,17,20,27], principal component analysis (PCA) [1,17,24], and cluster analysis (CA) [17,33,34]—have been widely used to determine the sources of heavy metals in the large-sized particles of street dust. These methods have been proven effective, however, their disadvantage so far has been that it has not been possible to quantitatively determine the contribution rate to the flux of each heavy metal from different sources, especially with respect to particles smaller than 100 um. The best of the multivariate statistical methods is known as the absolute principal component scores with multiple linear regression (APCS-MLR), which has become a recognized statistical model for quantitative pollution source allocation [1,17,33]. 

Studies on the concentration and distribution of heavy metals in street dust have demonstrated anthropogenic influence, which varies in different functional areas of cities, where different land use patterns apply. Different human factors in each functional area can release different heavy metals, leading to large differences in heavy metal content in the emissions from various functional areas [18,35,36]. Additionally, by applying geographic information system (GIS) mapping, the spatial distribution of heavy metal pollution can be linked to specific geographic patterns, such as industrial or heavy traffic areas, and population density. Therefore, in this study we used PCA, CA, and GIS mapping to conduct a detailed investigation on the heavy metal content of the small particles (<74 μm) in Shijiazhuang street dust.

The purposes of the research reported here were as follows:

1. To determine the concentrations of seven heavy metals (Cd, Cr, Cu, Hg, Ni, Pb, and Zn) in Shijiazhuang street dust.
2. To study heavy metal spatial distribution characteristics in the study area, using its ring roads as markers.
3. To assess the pollution level and enrichment degree for the targeted heavy metals by using the index of geoaccumulation (Igeo), and enrichment factor (EF) methods.
4. To apply multivariate statistical analysis to determine street dust heavy metal sources in the study area, and to quantify the relative contribution from each source, using the APCS-MLR model.
5. To identify heavy metal pollution control measures based on the metal distribution characteristics and source apportionments identified in this study.

The overall objective has been to determine and evaluate heavy metal pollution areas in Shijiazhuang, and to establish a comprehensive geochemical data interpretation method. The intent is to provide a template for creating an important information base for health risk assessment and pollution prevention in the urban environment.

## 2. Materials and Methods 

### 2.1. Study Area

Shijiazhuang is located between 114°23′–114°42′ E longitude and 37°58′–39° N latitude. It is located in the central and southern part of the large Hebei Plain, China, north of the capital Beijing and the port city of Tianjin, east of the Bohai sea and the northern oilfield, west of the Taihang mountains, and adjacent to the national coal base in Shanxi Province. The city has a warm, temperate, continental monsoon climate. Spring is relatively dry, with a northerly or southerly wind. In summer (June–August), under the influence of warm, humid oceanic airflow, precipitation accounts for 63–70% of the annual average, while autumn, affected by high pressure systems from Mongolia, is sunny with less rain, with the wind turning NW in late autumn. In winter, the NW wind prevails, under the influence of cold Siberian high-pressure systems. The annual average temperature is 14.2 °C, the annual total precipitation is between 513.6 and 718.5 mm, and on average there are ~2200 h of sunshine annually. 

**Figure 1:**
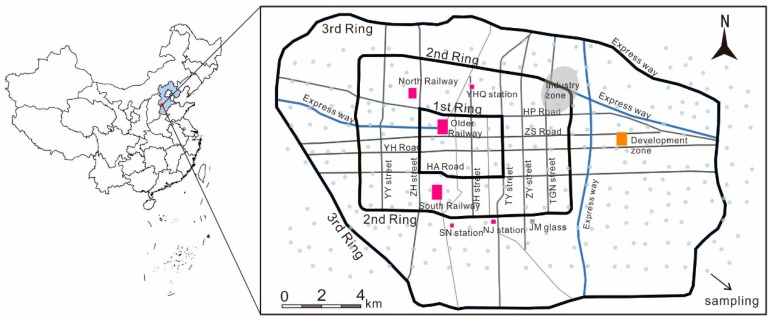
Study area location and map, showing the 1st, 2nd, and 3rd ring roads.

The research area mainly included the Shijiazhuang districts of Changan, Qiaodong, Qiaoxi, Yuhua, and Xinhua, encompassing a total area of 455.81 km², with a population of about 3.22 million. The study area includes three ring roads: The 1st ring road is composed of four main roads—Heping Road, Tiyu Street, Huaian Road, and Zhonghua Street—and is encircled by the 2nd ring road and the 3rd ring road, in sequence. The main E–W arterials are Heping, Zhongshan, Yuhua, and Huaian roads, and the principal N–S arterials are Youyi, Zhonghua, Pingan, Jianshe, Tiyu, Jianhua, Zhaiying, and Tangu streets, as shown in Figure 1.

### 2.2. Sample Collection

Sampling was conducted in clear and calm weather conditions from June to July, when there had been no significant rain or strong wind for at least a week beforehand. The sampling process was conducted to avoid interference from direct pollution sources. On-site, dust distribution characteristics of the study area were reviewed and, where it was considered to be evenly distributed, dust samples from each street were collected from 1 m² quadrats using a brush and plastic shovel at each location. In total, 234 street dust samples (particulate matter) were collected, with each sample weighing ~200 g. Sampling point locations were recorded with GPS positioning to log their longitude and latitude, and records were made of the surrounding environmental conditions and the sample number. The collected samples were packed into pre-labeled, polyethylene plastic bags, and returned to the laboratory for natural air drying at room temperature. After air drying, the samples were screened, using a 20 mesh nylon mesh, to remove large objects, including stones, leaves, cigarette butts, hair, etc.

### 2.3. Chemical Analysis 

During sample pretreatment, all containers were soaked in 30% nitric acid for 48 h, then washed with ultra-pure water and dried in an oven at low temperature. Dust samples were digested with HNO_3_-HF-HCl, after passing through a 74 μm nylon sieve. In the digestion process, blank samples, repeat samples, and national standard samples were identified, for quality control, in compliance with soil component analysis standards GBW07406 (GSS-6) and GBW07427 (GSS-13). The accuracy of the results was validated using blanks and standards, allowing the standard soil sample error to be controlled to within 5%. Hg was determined with atomic fluorescence spectrometry (Beijing HaiGuang instrument co., LTD, Beijing, China), with detection limit of 5 ng/g. Graphite furnace atomic absorption spectrometry (Agilent Technologies Inc., California, USA) was used for Cd analysis, with analytical limit of 0.02 μg/g. Pb, Cu, Zn, Ni, and Cr were analyzed using X-ray fluorescence (PANalytical B.V., Almelo, Netherlands). X-ray fluorescence has been applied to street dust analysis by a large number of scholars [1,13,16,17], with detection limits of 2, 1, 3, 2, and 3 μg/g, respectively.

### 2.4. Enrichment Factor 

Enrichment factor (EF) is an important index quantifying interference to the natural environment caused by anthropogenic activities. By comparing heavy metal element concentrations in samples with background levels, the anthropogenic influence status in the surface environment can be determined [1,37]. The reference element is the main constituent element in the reference substance and is ubiquitous in the sample: Elemental Fe was selected as the reference in this case, allowing Shijiazhuang street dust heavy metal EF to be calculated, as shown in Equation (1).

EF = (Si/Sn) sample/(Bi/Bn) reference(1)

where, Si represents the concentration of heavy metal element i, Sn is the concentration of Fe in the street dust sample, Bi is the heavy metal concentration of the reference sample, and Bn is the Fe concentration of the reference sample. If the element EF is close to 2, it can be considered that the element showed no enrichment compared to the soil source and is therefore mainly composed of soil particles. Values in the range 2–5 show moderate enrichment, indicating that elements are influenced by human activities in addition to soil sources [1,37]. If the element enrichment factor is >5, this is an indication primarily of significant enrichment. In this study, the reference values for heavy metal elements in the study area were determined by reference [38], which could better reflect the superposition of heavy metal pollution in street dust.

### 2.5. Geoaccumulation Index

The geoaccumulation index (Igeo) can be used in both soil and in all kinds of dust [1,17,38]. In this study, Igeo has been referred to as the Muller Index, as the indicator [39] applied to evaluate the heavy metal pollution characteristics of Shijiazhuang street dust. The Igeo formula can be expressed as shown in Equation (2).

(2) $$I_{geo}=log_{2}\left(Si/1.5Bi\right)$$

where, *Si* is the concentration of element *i* in the sample, *Bi* is the background reference concentration, 1.5 is the modified index, which is usually used to characterize sedimentary characteristics, petrogeology, and other influences. *I_geo_* is divided into 7 levels: *I_geo_* < 0, indicates no pollution; 0 ≤ *I_geo_* < 1, no pollution to moderate pollution; 1 ≤ *I_geo_* < 2, indicates moderate pollution; 2 ≤ *I_geo_* < 3 indicates moderate to strong pollution; 3 ≤ *I_geo_* < 4 indicates strong pollution; 4 ≤ *I_geo_* < 5, represents pollution levels between strong and extremely strong; when *I_geo_* ≥ 5 and the pollution level is 6, extremely strong pollution is present. The cumulative index not only considers the influence of natural background values on geological processes, but also pays close attention to the impact of external factors on heavy metal pollution. Therefore, a change in the index not only reflects the nature of the heavy metal distribution characteristics, but also distinguishes the impact of external factors on the environment—making it an important indicator of external, anthropogenic factors. 

### 2.6. APCS-MLR and CA

The street dust heavy metal sample data were analyzed using principal component analysis (PCA) and cluster analysis (CA)—techniques previously applied with success in related research [1,17,24,33,34]. PCA is a simplified data set technique in statistics processing and was chosen as it could provide deep insight into the relationships between heavy metals in street dust. The variables observed in this study were subjected to variance maxima rotation and Kaiser standardization, in an effort to identify the possible sources of heavy metals in particulates smaller than 74 µm in Shijiazhuang street dust. In the CA, Ward’s method—which divides observed variables into more mutually exclusive clusters and augments the PCA results—was used, with Euclidean distance applied to measure inter-cluster distances between similar variables.

PCA and CA allowed us to obtain useful qualitative information on the sources of street dust heavy metal contaminants in small (<74 um) particles. In order to render these outcomes quantitative, APCS-MLR was then used—allowing actual contribution rates from each source to be estimated. The APCS-MLR receptor model can be expressed as shown in Equation (3).

(3) $$\mathrm{Si}=\overset{n}{\underset{j=1}{\sum }}PCS_{j}\times b_{ji}+b_{i}$$

where Si is the heavy metal content, and *b_i_* is the multiple linear regression constant for each heavy metal source. Symbol *b_ji_* is the regression coefficient for heavy metal from *j* source, *n* is the number of sources, and *PCS_j_* is the value of rotation factor *j* for processed samples, calculated by subtracting the artificial sample factor score from the real sample factor score. *PCS_j_* × *b_ji_* is the contribution of source *j* to Si. The mean value of *PCS_j_* × *b_ji_* is the absolute contribution from source *j* for all investigated samples to heavy metal *i*. Accuracy was verified using the real value divided by the predicted value (the value of P calculated by receptor model) (S/P) [1].

### 2.7. Statistical Methods

The sources of—and relationships between—heavy metal concentrations in Shijiazhuang street dust were studied using data statistics (including min, max, SD, etc.), PCA (Varimax, with Kaiser Normalization), and CA (Ward’s method), implemented using IBM SPSS Statistics 24.0. Spatial distributions were 3-D (three dimension) mapped as predicted by Windows Surfer 13.0, using the kriging method (Wiener–Kolmogorov prediction). The EF and Igeo box-plot was created with Origin 2018 64 bit, and the source apportionment was created using Excel 2016.

## 3. Results and Discussion

### 3.1. Heavy Metal Concentrations

Statistical analysis results for Shijiazhuang street dust heavy metal concentration are shown in Table 2, with Hebei soil values [38] taken as reference values for this study. Arithmetic average concentrations of Cd, Hg, Cr, Pb, Zn, Cu, and Ni, were 1.86, 0.29, 131.7, 154.78, 496.17, 91.06, 40.99 mg/kg⁻¹, respectively, which were higher than the corresponding local soil reference values (which were respectively 0.09, 0.04, 68.3, 21.5, 71.9, 21.8, 30.8 mg/kg⁻¹). Zn, Hg, Pb, and Cd concentrations in Shijiazhuang street dust were particularly high, especially the Cd. The maximum concentration of Cd was 82.5 mg/kg⁻¹, which was 917 times the reference value, with these values suggesting overall that the metals came from human sources. In addition, the average concentrations of Hg, Pb, and Zn were five times higher than the corresponding reference values, with these multiples clearly higher than those exhibited by the other elements.

Variable coefficient value reflects the heterogeneous distribution trend in the environment. Where the coefficient of variation exceeds 0.7, which was particularly notable for Cd, Cr, Pb, Cu, and Ni, showing high variation, it was showing clearly that those heavy metals were demonstrating anthropogenic influence. Skewness value and kurtosis value indicate where those heavy metal element concentrations do not obey normal distribution—and this applied particularly to Cd, Cr, and Ni, whose kurtosis values were very high, indicating they were highly anomalous. The skewness results showed strongly that all the measured heavy metals in street dust were normally inclined towards lower concentrations, illustrated by the results showing that the median concentrations were lower than the mean concentrations [1]. 

We compared the heavy metal content of street dust from Chinese and other sources (Table 1), and it can be seen that the Shijiazhuang concentrations of the seven measured heavy metals were generally higher than elsewhere, including the Chinese cities of Xi’an, Beijing, Changchun, and Wuhan, and Toronto (Canada)—and that this particularly applied with respect to Pb, Zn, Cd, and Hg.

**Figure 2:**
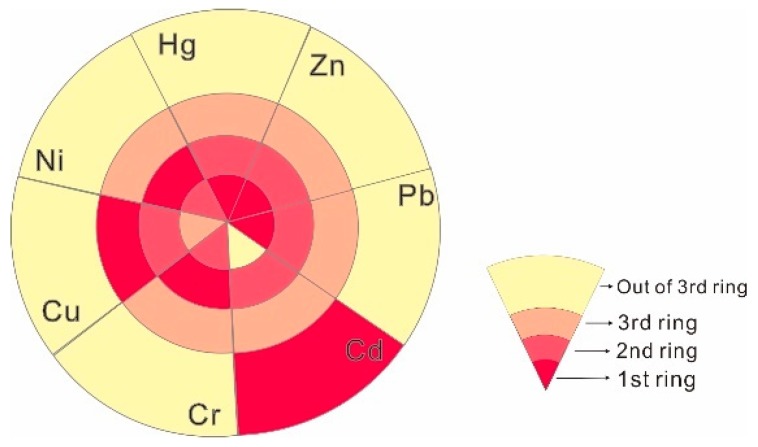
Distribution trend map for heavy metal concentration (darker = higher) in each Shijiazhuang ring.

According to the population, industry, traffic, and other structural characteristics applicable to each of the Shijiazhuang rings, the population density and traffic pressure hierarchy is 1st ring > 2nd ring > 3rd ring > outside of the 3rd ring, while the industrial level is 2nd ring > 3rd ring > outside 3rd ring > 1st ring. Dust samples from each ring were analyzed (Table 2), and it was found that the average concentrations of Hg, Pb, and Zn gradually decreased from the 1st ring to outside of the 3rd ring (Figure 2), which correlated with the population density and traffic pressure characteristics for the main urban area. Cd, Ni, and Cr maximized in the 2nd ring, which is the location of the most industry. The 3rd ring has the lowest level of external industrial elements, and also the lowest levels of Hg, Zn, Pb, Cr, Cu, and Ni. The concentration distribution of Cd was exceptional, and it is suggested that Cd pollution should be the subject of more detailed investigation in the future, including use of Cd isotopes to find specific pollution sources.

### 3.2. Spatial Distribution 

**Figure 3:**
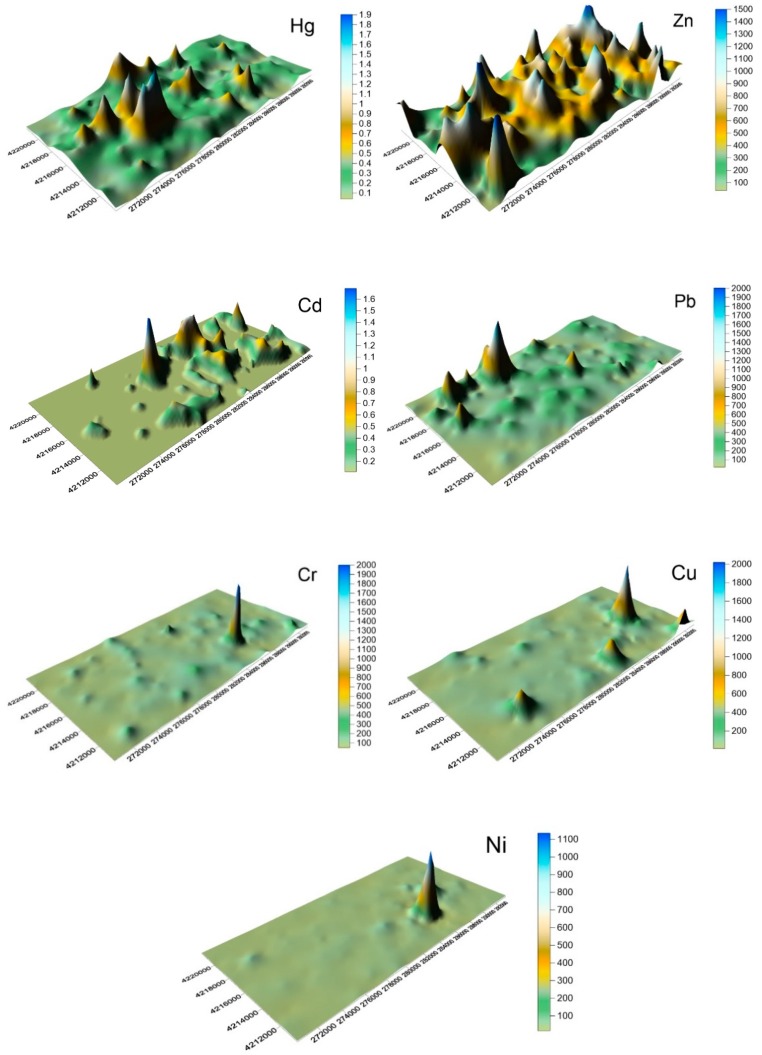
3-D (three dimension) heavy metal distribution in Shijiazhuang street dust.

Heavy metal street dust spatial distribution characteristics can reflect possible heavy metal sources [2,3,4,5,17,33,35]. In this study, Surfer software, incorporating the ordinary kriging interpolation method, was used to map the distribution of Ni, Cr, Cu, Hg, Cd, Pb, and Zn in street dust. The spatial distribution illustration in Figure 3 gives an intuitive reflection of the heavy metal distribution in Shijiazhuang, and shows that across the city, there were great differences in sample content. 

The Figure 3 shows that Hg, for example, had a wide distribution range, including the area surrounding the thermal power plant (the area between Youyi Street and Zhonghua Street, with Huaian road as its center). In addition, the area close to the city wireless power plant to the north of the 2nd ring road also exhibited relatively high values. 

The Zn concentration in the study area was also generally high and was distributed through industrial areas and commuter hotspots. The Ni focus was in the development zone, with other areas showing lower Ni level. The highest Cr values came from near the Jimei glass factory, while lower values were distributed in the same areas as the low Ni results. High Cu concentrations were located near the development zone and at commuter stations with high traffic flows. Higher Pb concentrations were found across a relatively large area, mainly at the intersection of North station, Zhongshan Road, and Tiyu Street. The north pharmaceutical factory area, the railway station, and the downtown southern station were also higher, potentially reflecting nearby traffic and its emissions. Cd meanwhile also exhibited raised levels across large areas, with peaks near the coking plant in the north, and other areas closely related to the existence of metals processing factories.

### 3.3. Enrichment Factors (EFs) and the Geoaccumulation Index (Igeo)

**Figure 4:**
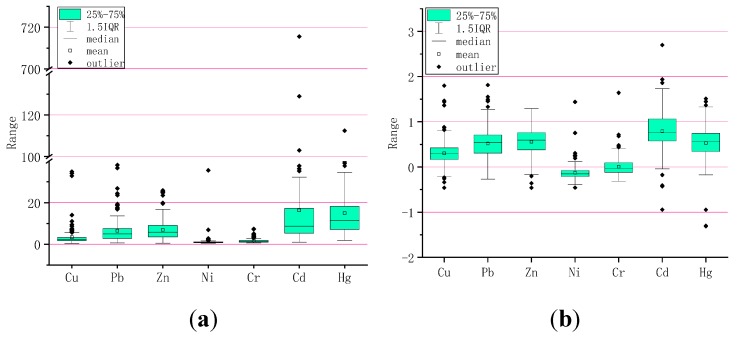
(**a**) Enrichment factors (EF) and (**b**) Geoaccumulation index (Igeo) boxplots for heavy metals associated with dust particles smaller than 74 μm.

The EF results for Shijiazhuang street dust heavy metals are shown in Figure 4. Average Ni and Cr values were below two in all samples, indicating that the concentrations of these heavy metals were just slightly enriched. The EF mean for Cu was generally (55.98% of samples) within the two to five range, indicating that this element was the subject of moderate enrichment in the study area. The average EFs of Pb (6.53) and Zn (6.93) were in the 5–10 range (41.03% of Pb and 41.88% of Zn), indicating that the Pb EF was a little less than Zn, and that both suffered strongly moderate enrichment in the study area.

Cd (16.3) and Hg (14.99) average EF values were both >10, exhibiting significant enrichment. Breaking these figures down, it could be seen that 18% and 14.53%, respectively, of all Cd and Hg street dust samples showed moderate enrichment levels (from two to five), 34.62% and 27.78% showed strongly moderate enrichment (from 5 to 10), particularly Cd, while 43.16% and 57.69% showed EF values >10, representing significant enrichment.

The figures show that heavy metals in Shijiazhuang street dust exhibited various enrichment levels. Cd and Hg showed significant enrichment in close to half of all samples, followed by Zn and Pb, which were also strongly enriched in all the samples. Cu showed slight enrichment, although less than Cd, Hg, Zn, or Pb. Ni and Cr showed the lowest enrichment levels.

Figure 4 also shows geoaccumulation index results for Shijiazhuang street dust heavy metals. It can be seen that the geoaccumulation indexes for Cu, Pb, Zn, Ni, Cr, Cd, and Hg in the study area ranged between -0.46–1.8, -0.27–1.81, -0.46–1.29, -0.46-1.44, -0.32–1.64, -0.94–2.7, and -0.88–0.71, respectively. Cd and Hg had large changes, and the overall geoaccumulation index hierarchy was Cd (0.79) > Zn (0.56) > Hg (0.53) > Pb (0.52) > Cu (0.3) > Ni (0.13) >Cr (0.01). Apart from Ni and Cr, the pollution degree indicated for the heavy metal elements (Pb 8.12%, Zn 5.98%, Cd 3.42%, Hg 6.41%) was level 1, indicating the status of ‘slightly polluted’.

### 3.4. Source Apportionment

#### 3.4.1. Correlation Coefficient Analysis

**Figure 5:**
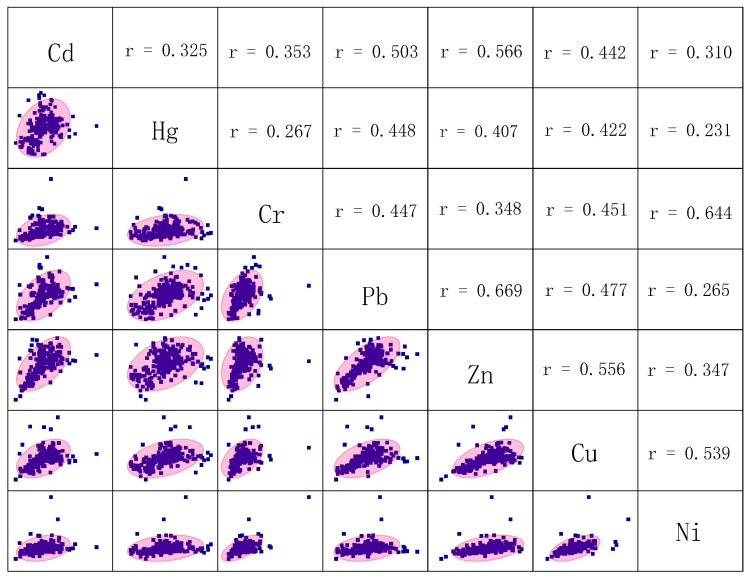
Correlation coefficients (95% confidence interval) and scatter diagrams for heavy metal matrixes in Shijiazhuang street dust (particles smaller than 74 µm).

Correlation analysis on heavy metals in study area street dust was conducted (Figure 5) using the Pearson method, after logarithm transformation and with data fitted to normality, or close to normality. Some heavy metal pairs—Ni–Cr, Zn–Cd, Pb–Zn, Cu–Ni, and Pb–Cd—showed positive correlations at the correlation coefficient > 0.5, at *p* < 0.01. Cd, Pb, and Zn were significantly positively correlated with each other, which may be because they are associated with similar sources—industrial activities or heavy traffic—with Pb in particular most likely associated with heavily trafficked parts of the study area. Hg showed relatively weak, positive correlations with Cd, Pb, and Zn, and very weak, positive correlations with Cr, Cu, and Ni. 

#### 3.4.2. PCA and CA

**Figure 6:**
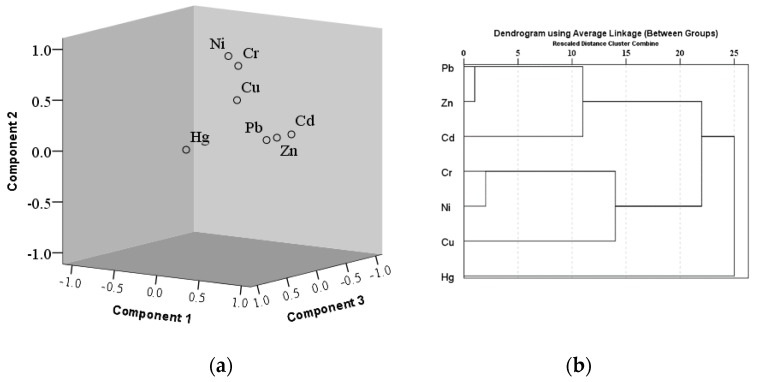
Analysis of seven heavy metals in Shijiazhuang street dust: (**a**) Results from PCA in 3-D space, and (**b**) dendrogram results from applying the Ward method CA.

To eliminate negative influence on the order of magnitude of heavy metal elements, the PCA was carried out after standardizing. The result of the Kaiser–Meyer–Olkin Test for Sampling Adequacy was 0.797, and the extraction of Communalities were >0.6. PCA was a suitable method by which to source apportionment, as a next step. Three eigenvalues greater than one were obtained by rotation, as shown in Table 3. Meanwhile, PCA in 3-D (three dimension) space and CA were summarized in Figure 6. The total cumulative contribution rate of the corresponding three principal components was 76.84%. The first principal component (PCA1) consisted of Cd, Pb, and Zn, with a contribution rate of 31.84%; the second principal component (PCA2) consisted of Cr, Cu, and Ni, with a contribution rate of 27.82%, and the third principal component (PCA3) was Hg, with a contribution rate of 17.16%. 

Considering the spatial distribution of Cd, Pb, and Zn, which were distributed in PCA1, based on the load value of rotation, Cd showed a northern high hotspot in the vicinity of a coking plant, the higher Zn concentration was in the urban traffic area, while Pb exhibited high value areas in the south by the city bus terminal and an industrial glass factory. These observations indicated that the visible principal component PCA1 had traffic and industry as its main source, which we have defined here as a mixed source. 

The PCA2 group, including Cr, Cu, and Ni, were mainly from natural sources, according to the enrichment factor and the geoaccumulation index.

The Hg load value was relatively larger than those of other heavy metals, in PCA3. Looking at the spatial distribution of Hg concentration and enrichment, it has a wide distribution of high concentration and enrichment, which fits with its past identification as an important indicator of coal combustion [40,41]. Hence, Hg was defined as a burning coal group.

**Figure 7:**
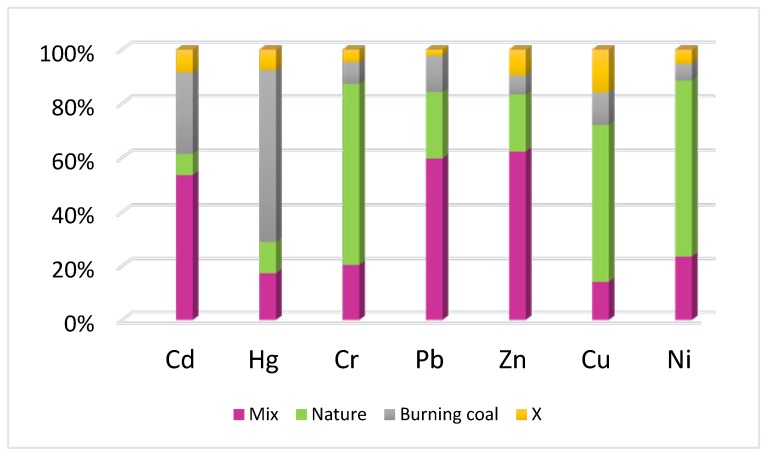
Heavy metal percentage contribution from different sources.

The APCS-MLR receptor model can quantitatively calculate each source’s contribution rate to each heavy metal—and the results from this analysis have been listed in Table 3. Using the APCS-MLR shown in Fig. 6, the contribution rates from the source groups were calculated, with results indicating that the contribution rate of PCA1 to Cd, Pb, and Zn were 53.55%, 59.7%, and 62.25%, respectively. PCA2 contributed > 50% to Cu, Cr, and Ni, accounting for 58.1%, 66.91%, and 65.09%, respectively. The contribution rate from PCA3 to Hg was 63.38%; PCA3 also contributed 30.09% to Cd, showing that Cd, as the second largest source in PCA3, was also sourced from coal combustion. To confirm the validity of this method, the calculations showed that the contribution rate from unknown sources (X) to Pb, Zn, Cr, Ni, Hg, and Cd emissions was < 10% for all cases, with the exception of Cu, for which the contribution from unknown sources was 15.84% (Table 4 and Figure 7). 

The APCS-MLR results have been presented in Table 4, where it can be seen that correlation coefficient values for all measured heavy metals in street dust were >0.7, indicating that the predicted values agreed well with the measured values [42,43]. The higher the correlation coefficient, the more reliable the prediction model [42,44]. The S/P ratio was determined for all heavy metals (Table 4), indicating that the source apportionment results for Shijiazhuang street dust heavy metals, determined using the APCS-MLR receptor model, were valid.

### 3.5. Sustainable Management

Considering the distribution of heavy metal concentration in street dust, and the structural characteristics of each ring, we suggest three major aspects that should be considered when promoting sustainable management in the study area.

#### 3.5.1. Industrial Enterprises Accelerated Transformation and Upgrading

At present, there are pharmaceutical factories, steel mills, thermal power plants, and other heavy emitters in the 2nd ring road area. We suggest that the government imposes direct administrative control on enterprises that cause serious pollution, in the form of mandatory orders, and formulates strategies for heavy polluters to be moved out of the main urban areas to avoid ongoing pollution and the associated human health impacts there. The strategy will be to eliminate outdated production processes and equipment—and technologies that consume large amounts of energy and materials—and to support industries that save energy and resources vigorously. The strategy will also encourage enterprises that render toxic pollutants harmless, by treatment and recycling, in order to reduce the environmental load.

#### 3.5.2. Improve the Urban Street Environment

In order to avoid secondary pollution, that is, re-suspension of dangerous and polluting dust, when dry, by the wind, a system of street water sprinklers, for both sides of the streets, should be implemented. The idea is to clean dust away from the main streets, especially in the strong wind period of the spring monsoon. Dust polluting passenger commuter nodes and industrial factories should be regularly removed by cleaning to eliminate the source of dust as much as possible. Another, better way to cure the pollution is to plant more dust-absorbing green plants. This is allowed to happen for technical and managerial reasons. The local government should address this through development of policies on street environment that consider heavy metal loads and should include a program of public awareness and involvement in policy decision-making as part of the policy development process.

#### 3.5.3. Relieve POPULATION pressure 

The population density of central Shijiazhuang is 7,064 people / km², which far exceeds the first level of population density classification (>100 people/km² in densely populated areas). The population concentration that extends from the western part of the city to downtown is very clear [45]. We have confirmed in this study that the level of heavy metal pollution gradually increases from the 3rd ring to the 1st ring, and the increasing population density directly contributes to this pollution through the increased number of cars. In 2017, the number of cars in Shijiazhuang was 2.47 million, causing serious congestion in the main street, and aggravating the heavy metal pollution situation in the air and street dust. It is suggested that the government develops a population alleviation policy, aimed at reducing population pressure in the study region.

## 4. Conclusions

A detailed investigation of heavy metals in street dust particles smaller than 74 µm was carried out in Shijiazhuang, China, and various heavy metals—particularly Hg, Cd, Pb, and Zn—were found to be present in concentrations quite different to background values. EF and Igeo results indicated that, with the exceptions of Ni and Cr, heavy metal elements enrichment in the street dust was moderately significant, at the pollution level of class 1, which is slightly polluted. Moreover, the Hg, Cd, Pb, and Zn concentrations were consistent with the urban population, traffic, and industrial structure base, and with the spatial distribution of main urban areas in relation to distance from the city center, as represented by the three ring roads. These four heavy metals (Hg, Cd, Pb, and Zn) were confirmed to be from anthropogenic sources. In addition, this study implied that Hg, Cd, Pb, and Zn concentrations in street dust has highly contributed to local (non)exhaust emission. 

The heavy metals in street dust of study area were divided into 3 principal source groups by PCA, including natural sources; mixed transportation and industry sources; burning coal source. The APCA-MLR results have indicated that the street dust particles are the major sources of pollutants, and thereby, this study would provide important information in developing policies for strategic management aimed at prevention and control of heavy metal pollution in the urban environment, especially in densely populated areas.

## Figures and Tables

**Table 1:** Heavy metal distribution in street dusts of different cities (mg·kg^−1^).

Country	City	Pb	Zn	Cu	Ni	Cr	Cd	Hg	Size (μm)	Reference
China	Shijiazhuang	154.78	496.17	91.06	40.99	131.70	1.86	0.29	<74	this study
	Xi’an	97.4	169.2	46.6	29.3	177.5	-	-	<74	[17]
	Beijing	105	222	69.9	25.2	84.7	0.72	-	<125	[18]
	Changchun	93.6	465.35	68.4	-	95.98	0.62	0.24	<74	[19]
	Lanzhou	62.65	296.92	72.97	-	62.14	-	-	<74	[20]
	Wuhan	102.6	224.2	62.1	27.7	75.3	-	-	-	[21]
	Shanghai	295	734	197	84	159	1.23		-	[22]
	Chengdu	375	1117	244	88.1	114	4.4		<74	[22]
	Guizhou	67.81	185.98	129.80	61.07	131.23	0.62	0.38	<149	[23]
Korea	Seoul	144	795	396	-	151	-	-	-	[17]
	Shihwa	612	1824	992	164	498	2.22	0.08	-	[24]
Japan	Tokyo	264	2200	-	43	73.9	1.4	-	<50	[25]
	Osaka	229	1070		19	35.1	1.04	-	<50	[25]
	Kyoto	156	2250		76.7	54.8	1.03	-	<50	[25]
India	Delhi	120.7	284.5	191.7	36.4	148.8	2.65	-	<74	[26]
Canada	Toronto	182.8	200.3	162	58.8	197.9	0.51	-	<150	[27]
England	Birmingham	48	534	467	41.1	-	1.62	-	-	[28]
Greece	Kavala	301	272	124	58	196	0.2	0.1	<63	[29]
Ghana	Accra	58.66	161.43	48.25	15.88	166.80	-	-	<40	[30]
Egypt	Cairo	234.6	638.4	-	25.38	36.95	0.82	-	<125	[31]
Nigeria	Jos	61	72	56.5	1.23	2.0	1.54	-	-	[32]

**Table 2:** Statistical analyses of Shijiazhuang street dust heavy metal content (unit: mg/kg).

*n* = 234	Cd	Hg	Cr	Pb	Zn	Cu	Ni
Mean	1.86	0.29	131.70	154.78	496.17	91.06	40.99
Median	0.98	0.22	95.90	113.00	424.50	65.00	32.55
SD	5.56	0.27	292.44	199.59	347.27	162.68	82.91
Variable Coefficient	2.99	0.96	2.22	1.29	0.70	1.79	2.02
Skewness	13.32	3.19	14.32	5.85	1.62	9.08	14.28
Kurtosis	192.36	12.71	213.64	46.56	3.56	97.82	211.87
Range	82.39	1.912	4437.60	2114.50	2074.70	2043.60	1256.90
Minimum	0.11	0.04	49.40	17.50	37.30	11.40	16.10
Maximum	82.50	1.95	4487.00	2132.00	2112.00	2055.00	1273.00
Background	0.09	0.04	68.30	21.50	71.90	21.80	30.80
**Rings (Samples)**	**The mean heavy metals values for samples from each ring**						
1st (15)	1.26	0.40	110.11	191.47	673.67	72.69	39.73
2nd (90)	2.65	0.36	169.54	175.85	507.54	95.83	48.76
3rd (104)	1.35	0.25	107.88	157.72	477.84	102.20	36.83
out of 3rd (25)	1.43	0.17	96.63	72.86	444.63	47.88	32.46

**Table 3:** Total variance explained and component matrix ^a^ of heavy metals in this study.

Components	1	2	3
Cd	0.822	0.201	0.012
Hg	0.224	0.123	0.934
Cr	0.246	0.827	0.085
Pb	0.754	0.186	0.333
Zn	0.817	0.202	0.248
Cu	0.437	0.552	0.379
Ni	0.124	0.910	0.079
Eigenvalues	2.229	1.948	1.202
% of Variance	31.842	27.824	17.175
Cumulative %	31.842	59.666	76.841

Extraction method: Principal component analysis. Rotation method: Varimax with Kaiser normalization. ^a^ Rotation converged in 4 iterations.

**Table 4:** Contribution (%) by pollution source to each heavy metal in Shijiazhuang street dust.

Elements	Mixed	Natural	Burning Coal	X	R^2^	S/P
Cd	53.55	7.84	30.09	8.51	0.706	1.00
Hg	17.33	11.62	63.38	7.67	0.933	1.00
Cr	20.42	66.91	7.98	4.68	0.747	1.00
Pb	59.70	24.61	13.41	2.27	0.676	1.00
Zn	62.25	21.17	6.95	9.62	0.768	1.00
Cu	14.13	58.01	12.01	15.84	0.678	1.00
Ni	23.52	65.09	6.11	5.28	0.753	1.00
